# *Acorus tatarinowii* Schott extract reduces cerebral edema caused by ischemia–reperfusion injury in rats: involvement in regulation of astrocytic NKCC1/AQP4 and JNK/iNOS-mediated signaling

**DOI:** 10.1186/s12906-020-03168-z

**Published:** 2020-12-09

**Authors:** Yu-Chen Lee, Shung-Te Kao, Chin-Yi Cheng

**Affiliations:** 1 Graduate Institute of Acupuncture Science, China Medical University, Taichung, 40402 Taiwan; 2 Department of Chinese Medicine, China Medical University Hospital 40447, Taichung, Taiwan; 3 Research Center for Chinese Medicine & Acupuncture, China Medical University, Taichung, 40402 Taiwan; 4 School of Chinese Medicine, College of Chinese Medicine, China Medical University, Taichung, 40402 Taiwan; 5 School of Post-baccalaureate Chinese Medicine, College of Chinese Medicine, China Medical University, Taichung, 40402 Taiwan; 6 Department of Chinese Medicine, Hui-Sheng Hospital 42056, Taichung, Taiwan

**Keywords:** *Acorus tatarinowii* Schott, Cerebral edema, Aquaporin 4, C-Jun N-terminal kinase, Intercellular adhesion molecule-1, Zonula occluden-1

## Abstract

**Background:**

This study aimed to evaluate the effects of the *Acorus tatarinowii* Schott [Shi Chang Pu (SCP)] extract administered at the start of 2 h of middle cerebral artery occlusion (MCAo), followed by 3 d of reperfusion, and to determine mechanisms involved in anti-edema effects in the penumbra of the cerebral cortex.

**Method:**

Rats were intraperitoneally administered the SCP extract at a dose of 0.25 g/kg (SCP-0.25 g), 0.5 g/kg (SCP-0.5 g), or 1 g/kg (SCP-1 g) at the start of MCAo.

**Result:**

SCP-0.5 g and SCP-1 g treatments effectively reduced the cerebral infarct size, ameliorated cerebral edema, reduced blood–brain barrier permeability, and restored neurological function. SCP-0.5 g and SCP-1 g treatments markedly downregulated the levels of glial fibrillary acidic protein, Na^+^-K^+^-2Cl^−^ cotransporter type 1 (NKCC1), aquaporin 4 (AQP4), phospho-c-Jun N-terminal kinase (p-JNK)/JNK, inducible nitric oxide synthase (iNOS), 3-nitrotyrosine, intercellular adhesion molecule-1 (ICAM-1), matrix metalloproteinase-9 (MMP-9), vascular endothelial growth factor-A (VEGF-A), and zonula occluden-1 (ZO-1) and upregulated ZO-3 expression in the penumbra of the cerebral cortex 3 d after reperfusion.

**Conclusions:**

SCP-0.5 g and SCP-1 g treatments exert neuroprotective effects against cerebral infarction and cerebral edema partially by mitigating astrocytic swelling and blood–brain barrier disruption. Moreover, the anti-cerebral edema effects of SCP extract treatments are possibly associated with the downregulation of astrocytic NKCC1/AQP4 and JNK/iNOS-mediated ICAM-1/MMP-9 signaling in the penumbra of the cerebral cortex 3 d after reperfusion.

## Background

The main pathological factors for ischemic stroke injury are inflammation and oxidative/nitrative stress, leading to the development of cerebral infarction and cerebral edema [1]. Accumulating evidence reveals that cerebral edema exacerbates cerebral injury and leads to the development of cerebral herniation, and these contribute to a high risk of death during ischemic stroke [2–4]. Based on the pathogenesis, cerebral edema is classified into two types: cytotoxic (intracellular) and vasogenic (extracellular) edema; edema results from water transport impairment in cells and blood vessels [2, 5]. Cerebral edema usually results from cytotoxic edema and subsequently progresses to vasogenic edema with a wide range of injuries [3].

Aquaporin 4 (AQP4), a member of the aquaporin family of membrane-bound proteins, is the most abundant water channel expressed in the perivascular astrocytic endfeet [6, 7]. AQP4 enables the passive transport of water across membranes according to osmotic gradients. Water transport occurs from blood vessels to astrocytes or the brain parenchyma through AQP4, which regulates blood–brain barrier (BBB) permeability, and this is the rate-limiting step for water influx into the brain [2, 7, 8]. During cerebral ischemia-reperfusion (I/R) injury, AQP4 plays a crucial role in the pathophysiology of cytotoxic edema [6]. Na^+^-K^+^-2Cl^−^ cotransporter (NKCC) exists in two isoforms: NKCC1 and NKCC2. NKCC1 is present in a wide range of tissues, whereas NKCC2 is predominantly found in the kidney. NKCC1 plays a role in regulating water and ion homeostasis in astrocytes [9]. In the early phase of cerebral ischemia, the levels of intracellular Ca^++^ and extracellular K^+^ are markedly increased in the ischemia area; subsequently, this leads to NKCC1-mediated Na^+^, K^+^, and Cl^−^ transport and accompanying water influx into brain cells, leading to astrocytic swelling, cytotoxic edema, and cerebral infarction [9, 10]. In addition, inhibition of NKCC1 expression in the ischemic region ameliorates brain swelling [8]. Under ischemic challenges, NKCC1- and AQP4-mediated astrocytic swelling leads to glutamate-mediated excitotoxicity, excessive nitric oxide (NO) production, and cytokine [such as interleukin (IL)-1β, IL-6, and tumor necrosis factor-α] release, which subsequently promote BBB disruption in the ischemic area, resulting in exacerbation of cerebral edema [11, 12]. Thus, the expression of glial fibrillary acidic protein (GFAP), a marker of reactive astrogliosis, in the ischemic region is positively correlated with the severity of cerebral edema [9, 13, 14].

Cerebral I/R injury induces the activation of c-Jun N-terminal kinase (JNK), one of the mitogen-activated protein kinases (MAPKs), which subsequently upregulates the expression of inducible nitric oxide synthase (iNOS); this generates excessive amounts of reactive oxygen/nitrogen species, leading to oxidative/nitrative stress in the ischemic area [15–17]. iNOS upregulation in ischemic brain cells, including endothelial cells, initiates the nitrosylation of tyrosine-containing proteins, forming 3-nitrotyrosine (3-NT, a marker of nitrative stress), which causes the opening of mitochondrial permeability transition pores, and exacerbates post-ischemic inflammation and BBB damage [18, 19]. Increased iNOS expression also enhances the synthesis of intercellular adhesion molecule-1 (ICAM-1) and matrix metalloproteinases (MMPs) (such as MMP-9) on endothelial cells [20, 21]. MMP-9 plays a pivotal role in extracellular matrix degradation and BBB disruption, causing hemorrhage and vasogenic edema [21]. The cerebral I/R injury-induced inflammatory response triggers BBB disruption and then increases BBB permeability, which upregulates vascular endothelial growth factor (VEGF) expression in the ischemic area [22, 23]. VEGF, a potent vascular permeability regulator, is positively related to the extent of vasogenic and cytotoxic edema in the acute phase of cerebral ischemic injury, whereas pharmacological inhibition of VEGF ameliorates vascular permeability and attenuates cerebral edema [3, 24]. Tight junction (TJ) proteins, which are major components of the BBB, include the integral membrane proteins occludin and claudins; these membrane proteins interact with the plasma membrane of endothelial cells, forming the TJ barrier and determining endothelial permeability [24, 25]. Cytoplasmic TJ accessory proteins, such as zonula occluden (ZO)-1, − 2, and − 3, stabilize the TJ by binding occludin to the cytoskeleton [26]. Studies have showed that augmented expression of ICAM-1 and MMP-9 disrupts the endothelial TJ promoting the infiltration of activated leukocytes into the brain parenchyma and triggering vasogenic edema [18, 23].

*Acorus tatarinowii* Schott, commonly known as Shi Chang Pu (SCP), is a well-known traditional Chinese medicine widely used to treat stroke, dementia, depression, seizure, and mental disorders. SCP is also an ancient herbal supplement, and it exerts anti-fatigue effects [27, 28]. Both α-asarone and β-asarone are the main active components in SCP [29]. α-Asarone enhances neural progenitor cell proliferation and differentiation into neuronal lineage cells and promotes neurofunctional recovery, as demonstrated in a mouse model of ischemic stroke [30]. β-Asarone can easily pass through the BBB, and exerts neuroprotective effects on ischemic injury by downregulating Beclin-1-dependent autophagy in an in vitro model of oxygen-glucose deprivation/reperfusion-induced PC12 cells [31]. In addition, α- and β-asarone have neuroprotective effects against cerebral I/R injury by reducing glutamate- or N-methyl-D-asparate-induced excitotoxicity in the ischemic area after cerebral ischemia [32]. Studies have reported that the combination of α- and β-asarone at low dosages produces synergistic neurotrophic effects and prevents cytotoxic-induced side effects in cell culture models [33, 34]. Moreover, β-asarone protects against cerebral I/R injury by stabilizing BBB integrity, and upregulating anti-oxidative and Na^+^-K^+^ ATPase activities in the ischemic area in the acute phase of middle cerebral occlusion (MCAo) [35]. During cerebral I/R injury, BBB permeability, oxidative stress, and Na^+^-K^+^ ATPase expression are closely associated with cerebral edema formation [36, 37]. Pharmacological restoration of BBB integrity and upregulation of Na^+^-K^+^ ATPase activity effectively ameliorate cerebral edema in rat models of transient MCAo [37, 38]. Based on the above findings, we speculate that SCP exerts anti-edema effects in a rat model of transient focal cerebral ischemia.

Thus, the aims of this study were to determine the anti-infarct, and anti-edema effects and mechanisms of the SCP extract administered at the start of MCAo, followed by 3 d of reperfusion.

## Methods

### Experimental animals

A total of 116 healthy male Sprague–Dawley rats (weight, 300–350 g; age, 8–9 wks) obtained from LASCO Co., Ltd. (I-Lan, Taiwan) were used in this study. The rats were housed in the animal room with the controlled conditions of 22–24 °C and 50–55% relative humidity under a 12/12-h light/dark cycle. Seven rats died during the experiments and the mortality rate was 6% in this study.

### Ethics statement

All surgical and experimental procedures were approved by the Institutional Animal Care and Use Committee of China Medical University (Permit Number: CMUIACUC-2018-304). The committee has recognized that the research methodologies and designs followed the Animal Protection Act by the Council of Agriculture, Taiwan. All surgical and experimental procedures were designed to avoid or minimize discomfort, distress, and pain to the animals.

### SCP extract preparation

Two grams of SCP extract powder (Chuang Song Zong Pharmaceutical Co., Ltd., Taiwan) was dissolved in 8 mL double-distilled water. After stirring for 2 min, the mixture was centrifuged at 1000×g at 4 °C for 10 min. The supernatant was collected in another tube, and the SCP aqueous extract was prepared at a final concentration of 0.125 g/mL.

### High performance liquid chromatography measurement of the indicators of the SCP extract

The standards including α-asarone (purity: 100%, National Institutes for Food and Drug Control, China) and β-asarone (purity: 95.52%, ChromaDex, USA) were accurately weighted and dissolved in pure methanol as the standard solutions. One and a half grams of the SCP extract powder was dissolved in 25 mL methanol, and the solution was centrifuged at 9000 rpm at 4 °C for 10 min. The supernatant was collected as the test solution. Subsequently, High performance liquid chromatography (HPLC) analytical procedures were performed, as described previously [39]. Briefly, twenty microliters of the standard or test solution was injected into a Waters HPLC system (Waters Alliance 2695 Separations Module, Waters Corp.). The mobile phase consisted of water (A) and acetonitrile (B). In gradient elution, the percentage of mobile phase A was decreased from 75 to 25% for 50 min, whereas the amount of mobile phase B was increased from 25 to 75%. The flow rate was set at 1.0 mL per min, and the total run time was 50 min. Absorbance was measured at 257 nm.

### Transient MCAo

MCAo was induced in the rats through the intraluminal suture occlusion technique, as previously described [39]. Briefly, the rats were anesthetized using isoflurane (5% for induction and 2% for maintenance). The rat’s head was fixed in a stereotactic frame, and the scalp was transversely dissected to expose the skull. Subsequently, a burr hole at a location 2.0 mm posterior and 2.5 mm lateral to the bregma was drilled into the skull for the assessment of blood flow in the right middle cerebral artery (MCA). After central neck dissection, the right external carotid artery (ECA) and internal carotid artery (ICA) were exposed. A 3–0 monofilament suture with a small round tip was gently inserted into the ICA through the ECA stump to occlude the origin of the MCA. Two hours after the induction of MCAo, the suture was carefully removed to allow reperfusion. During MCAo surgery, blood flow in MCA was assessed using Laser-Doppler flowmetry (DRT4, Moor Instruments Inc., USA). A decrease of greater than 80% in MCA blood flow in the phase of ischemia and an increase of greater than 60% in MCA blood flow in the phase of reperfusion verified the success of the MCAo model.

### Evaluation of neurological status

The neurological status of each experimental rat was evaluated using the modified neurological severity score (mNSS) at 1 and 3 d after reperfusion. The mNSS described previously [40] includes scores of motor, sensory, balance, and reflex tests. The neurological deficit score ranges from 0 to 18, with 0 as the normal condition and 18 as the maximal deficit score.

### Experiment A

#### Grouping

The rats were randomly divided into five groups: Sham (*n* = 5), Model (*n* = 5), SCP-0.25 g (*n* = 5), SCP-0.5 g (*n* = 5), and SCP-1 g (*n* = 5) groups. The rats in the SCP-0.25 g, SCP-0.5 g, and SCP-1 g groups were intraperitoneally administered with the SCP extract at the doses of 0.25, 0.5, and 1 g/kg, respectively, at the onset of MCAo. After 2 h of ischemia followed by 3 d of reperfusion, the rats were anesthetized with 5% isoflurane. Each anesthetized rat was placed in a plastic chamber and then underwent CO_2_ euthanasia (flow rate: 5.5 L/min) until 60 s after breathing had stopped. Finally, the brain was quickly removed. The rats in the Model group were subjected to the same experimental procedures as those in the SCP-1 g group, but the rats were administered with normal saline instead of the SCP extract. The rats in the Sham group were subjected to the same experimental procedures as those in the Model group, but MCA was not occluded.

#### Assessment of cerebral infarction

After completing neurological examination at 3 d of reperfusion, the rats were anesthetized with 5% isoflurane and then underwent CO_2_ euthanasia, and their brains were quickly removed. The fresh brains were sectioned into 2 mm-thick coronal slices, stained with 2,3,5-triphenyltetrazolium chloride (TTC; Merck, Germany) at 37 °C for 5 min, and then fixed with 4% paraformaldehyde overnight. In TTC staining, the white colored portion reveals the infarct area, whereas the dark-red colored portion reveals the non-infarct area. The percentage of the infarct area to the total coronal sectional area was calculated.

### Experiment B

#### Grouping

The rats were randomly divided into five groups: Sham (*n* = 9), Model (*n* = 7), SCP-0.25 g (*n* = 7), SCP-0.5 g (*n* = 7), and SCP-1 g (*n* = 8) groups. The experimental procedures are the same as those for Experiment A.

#### Measurement of water content in right cerebral hemispheres

After 3 d of reperfusion, the rats were deeply anesthetized with 5% isoflurane (3 min); subsequently, their brains were quickly removed, and the cerebella were discarded. The right (ipsilateral) and left (contralateral) hemispheres along the anatomic midline were dissected. Cerebral edema was determined by measuring water content in right cerebral hemispheres through the wet and dry weight method. Each right cerebral hemisphere was weighted (wet weight) and subsequently placed in the oven set at 100 °C for 24 h to obtain the dry weight. The percentage of water content in right cerebral hemispheres was calculated using the following formula: 100 × (wet weight-dry weight)/wet weight.

#### Measurement of BBB permeability in the ischemic cortex

BBB permeability was determined using the previously described Evans blue dye (EBD) extravasation method with certain modifications [41]. Briefly, after 3 d of reperfusion, the rats were anesthetized. Two percent EBD solution (E2129 Sigma-Aldrich) was injected intravenously into each rat at a dose of 100 mg/kg 2 h before sacrifice. In the sacrifice procedure, the rats were deeply anesthetized with 5% isoflurane (3 min) and transcardially perfused with 0.9% saline to remove all intravascular dye, and their brains were quickly removed and coronally sectioned from − 4.3 to + 1.7 mm of the bregma. The selected right (ischemic) cortex was homogenized with 1:2 volume of 50% trichloroacetic acid (T6399 Sigma-Aldrich) for 5 min and then incubated in a dark tube at 4 °C for 24 h. Subsequently, the homogenates were centrifuged at 10000×g for 25 min at room temperature (RT), and the final supernatants were diluted with 1:3 volume of 95% ethanol for the spectrophotometric analysis of EBD (620 nm excitation/680 nm emission wavelengths).

### Experiment C

#### Grouping

The rats were randomly divided into five groups: Sham (*n* = 5), Model (*n* = 5), SCP-0.25 g (*n* = 5), SCP-0.5 g (*n* = 5), and SCP-1 g (*n* = 5) groups. The experimental procedures are the same as those for Experiment A.

#### Western blot analysis

After 3 d of reperfusion, the rats were deeply anesthetized with 5% isoflurane (3 min) and then sacrificed, and their brains were quickly removed. The brain samples were separated into the cortical penumbra fractions, which were further divided into cytosolic and mitochondrial fractions, as described previously [39]. Subsequently, 15 μg proteins per well were loaded for 10% sodium dodecyl sulfate-polyacrylamide electrophoresis. After electrophoresis, the separated proteins were transferred onto nitrocellulose (NC) membranes, which were subsequently incubated with primary antibodies (Table 1) at 4 °C overnight. After washing, the NC membranes were incubated with appropriate secondary antibodies (1:5000 dilution) at RT for 1 h. Protein bands were revealed using the enhanced chemiluminescence plus reagent solution (GE Healthcare). The band intensity of target protein expression levels in relation to actin (or nonphosphorylated target protein) was calculated using ImageJ software (NIH, Bethesda, MD, USA).

**Table 1:** Primary antibodies used in this study

Species	Primary antibody	Western blotting Dilution	Immunohistochemistry (Immunofluorescence) Dilution	Source
Mouse	GFAP	1:1000	(1:100)	CST/#3670
Rabbit	NKCC1	1:1000	1:200 (1:100)	abcam/ab59791
Rabbit	phospho-JNK (p-JNK)	1:1000		CST/#9251
Rabbit	JNK	1:1000		CST/#9252
Rabbit	iNOS	1:250		abcam/ab15323
Rabbit	VEGF-A	1:1000		Proteintech/#19,003–1-AP
Rabbit	ZO-1	1:1000	1:200 (1:100)	abcam/ab96587
Rabbit	ZO-2	1:1000		CST/#2847
Rabbit	ZO-3	1:1000		abcam/ab191143
Mouse	3-NT		1:1000	abcam/ab61392
Mouse	AQP4		1:100 (1:50)	abcam/ab9512
Mouse	ICAM-1		1:100 (1:50)	abcam/ab171123
Rabbit	MMP-9		1:400	abcam/ab137867
Mouse	Actin	1:5000		NB/NB600–501

*CST* Cell Signaling Technology, *NB* Novus Biologicals

### Experiment D

#### Grouping

The rats were randomly divided into five groups: Sham (*n* = 4), Model (*n* = 4), SCP-0.25 g (*n* = 4), SCP-0.5 g (*n* = 5), and SCP-1 g (*n* = 4) groups. The experimental procedures are the same as those for Experiment A.

#### Immunohistochemical analysis

After 3 d of reperfusion, the rats were anesthetized with 5% isoflurane and then underwent CO_2_ euthanasia; subsequently, the rats were transcardially perfused with saline (0.9% NaCl). Their brains were quickly removed, embedded in the optimal cutting temperature compound, frozen, cut into 15 μm-thick coronal sections, and subsequently incubated with normal serum, as described previously [42]. The brain sections were incubated with primary antibodies (Table 1) at 4 °C overnight. The sections were subsequently incubated with appropriate secondary antibodies and avidin–biotin peroxidase complexes (Leica Biosystems Newcastle Ltd., UK). In all sections, immunopositive cells in the penumbra of the cerebral cortex were calculated in each of nine 400× magnification fields (1 mm^2^) under a light microscope (Axioskop 40, Zeiss). The adjacent sections from the Model group without primary antibody staining were used as negative controls.

#### Assessment of immunofluorescence double staining

The brain sections were incubated with 5% bovine serum albumin in phosphate buffered saline/Tween 20 (0.01%) (PBST) for 30 min at RT and were subsequently incubated with rabbit and mouse primary antibodies (Table 1) overnight at 4 °C. After washing three times with PBST, the brain sections were stained with DyLight 594-conjugated (1:200 dilution, Jackson ImmunoResearch) and 488-conjugated (1:200 dilution, Jackson ImmunoResearch) IgG secondary antibodies for 1 h at 37 °C. Immunopositive cells in the penumbra of the cerebral cortex were detected in each of nine 400× magnification fields (1 mm^2^) under a fluorescence microscope (CKX53, Olympus, Tokyo, Japan).

### Statistical analysis

All data are represented as the mean ± standard deviation. Statistical analysis of the data in this study was performed using one-way analysis of variance. Comparisons among experimental groups were made using the Scheffe test. A *P* value less than 0.05 indicated statistically significant differences.

## Result

### HPLC analysis of SCP extract

**Fig. 1:**
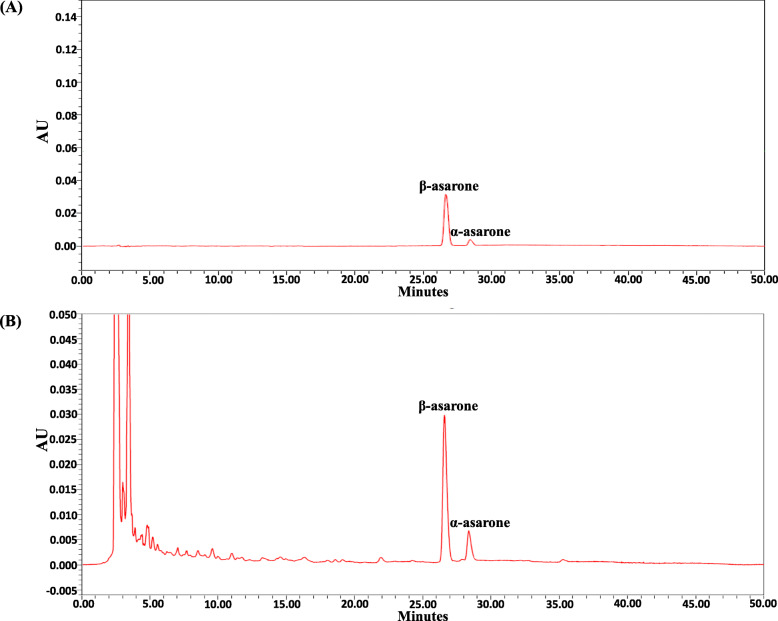
HPLC profiles of the **a** standard solution and **b** SCP extract solution. AU, Absorbance unit

For both the standard solution and SCP extract solution, the retention times of β-asarone and α-asarone were about 26.8 and 28.5 min, respectively. β-Asarone and α-asarone contents in the SCP extract were 0.16 and 0.01 mg/g, respectively (Fig. 1a and b).

### Effects of SCP treatments on cerebral infarction

**Fig. 2:**
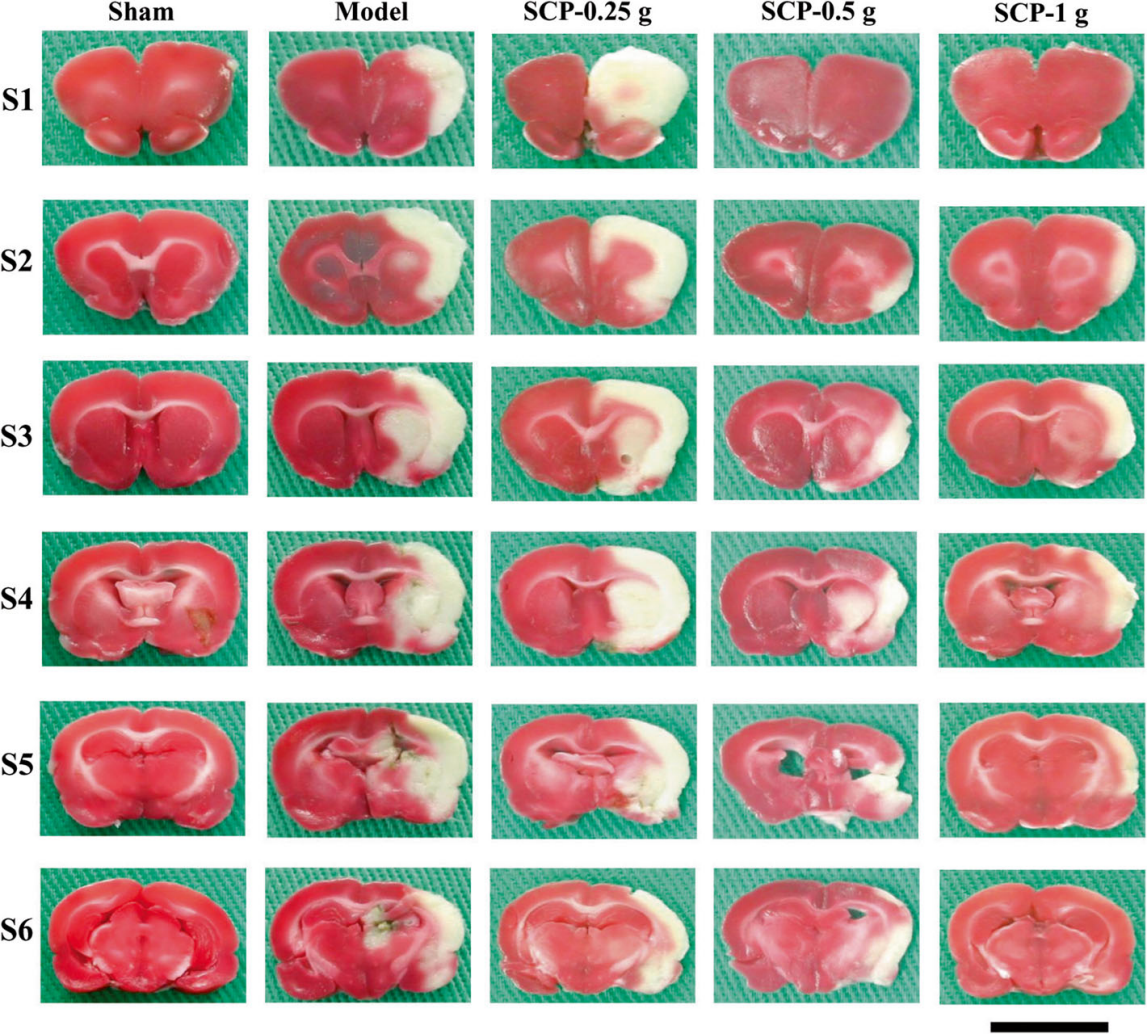
Representative coronal brain sections (S1–S6) among the experimental groups 3 d after reperfusion. In TTC stained brain sections, white color indicated the infarct area and dark-red color indicated the normal brain tissue. Scale bar = 1 cm

**Fig. 3:**
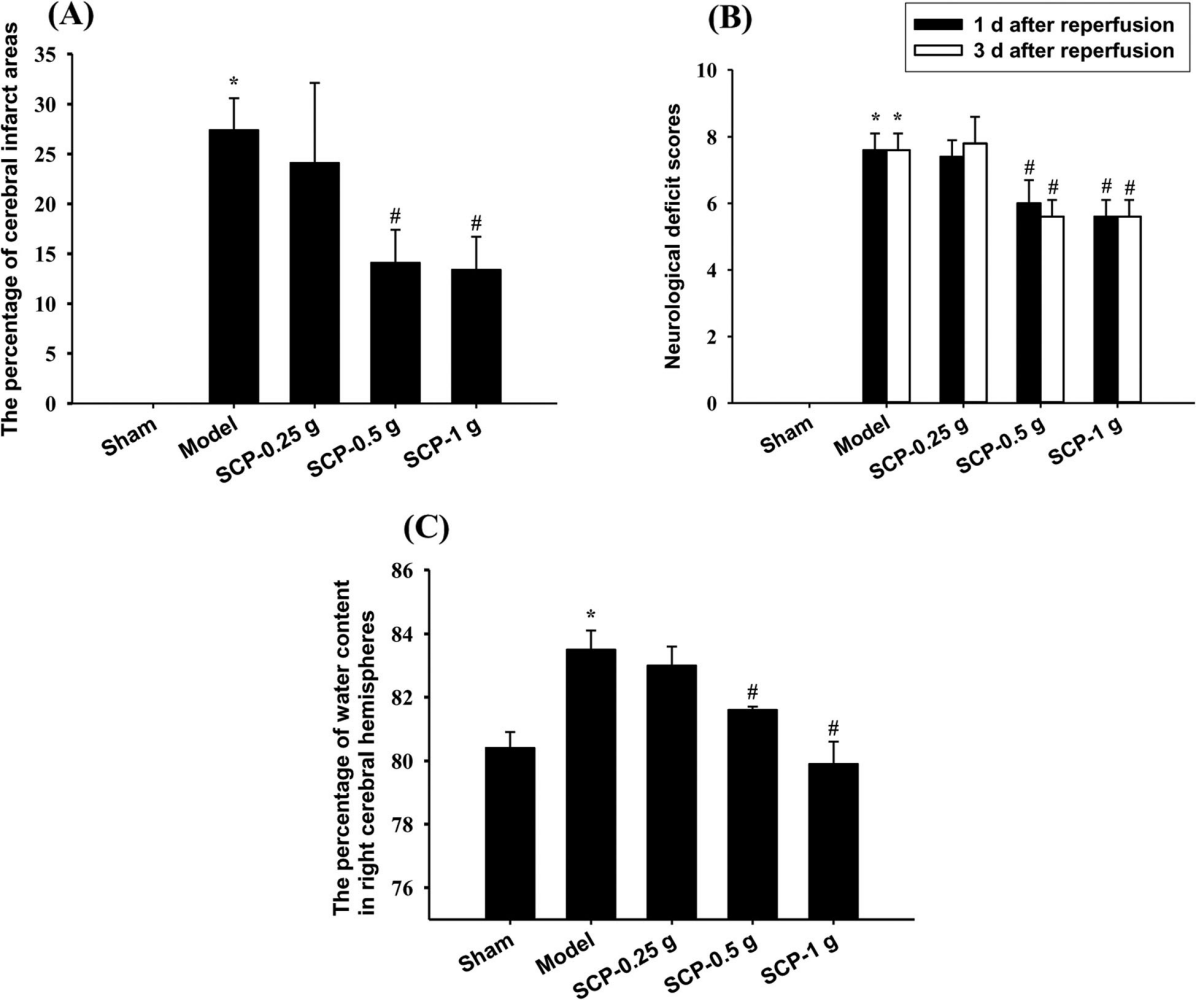
Effects of SCP-0.5 g and SCP-1 g treatments on cerebral infarction, neurological deficits, and cerebral edema 3 d after reperfusion. **a** The percentage of cerebral infarct areas in the Sham, Model, SCP-0.25 g, SCP-0.5 g, and SCP-1 g groups was evaluated 3 d after reperfusion. **b** Neurological deficit scores of the Sham, Model, SCP-0.25 g, SCP-0.5 g, and SCP-1 g groups were examined 1 and 3 d after reperfusion. **c** Water content in right cerebral hemispheres in the Sham, Model, SCP-0.25 g, SCP-0.5 g, and SCP-1 g groups was evaluated 3 d after reperfusion. Data are expressed as mean ± standard deviation. *P < 0.05 compared with the Sham group; #*P* < 0.05 compared with the Model group

After 3 d of reperfusion, the cerebral infarct areas were evaluated through TTC staining. The percentage of cerebral infarct areas markedly increased in the Model group compared with the Sham group (*P* < 0.05) and markedly decreased in the SCP-0.5 g and SCP-1 g groups compared with the Model group (both *P* < 0.05; Figs. 2 and 3a). No marked difference was found in the percentage of cerebral infarct areas between the Model and SCP-0.25 g groups (*P* > 0.05).

### Effects of SCP treatments on neurological status

After 1 and 3 d of reperfusion, the neurological deficit scores markedly increased in the Model group compared with the Sham group (both *P* < 0.05) and markedly decreased in the SCP-0.5 g and SCP-1 g groups compared with the Model group (all *P* < 0.05; Fig. 3b). However, no marked difference was found in the neurological deficit scores between the Model and SCP-0.25 g groups (*P* > 0.05).

### Effects of SCP treatments on water content in right cerebral hemispheres

Three days after reperfusion, water content in right (ipsilateral) cerebral hemispheres was measured using the tissue drying method. The percentage of water content in right cerebral hemispheres markedly increased in the Model group compared with the Sham group (*P* < 0.05) and markedly decreased in the SCP-0.5 g and SCP-1 g groups compared with the Model group (both *P* < 0.05; Fig. 3c). However, No marked difference was found in the percentage of water content in right cerebral hemispheres between the Model and SCP-0.25 g groups (*P* > 0.05).

### Effects of SCP treatments on BBB permeability in right cortical regions

**Fig. 4:**
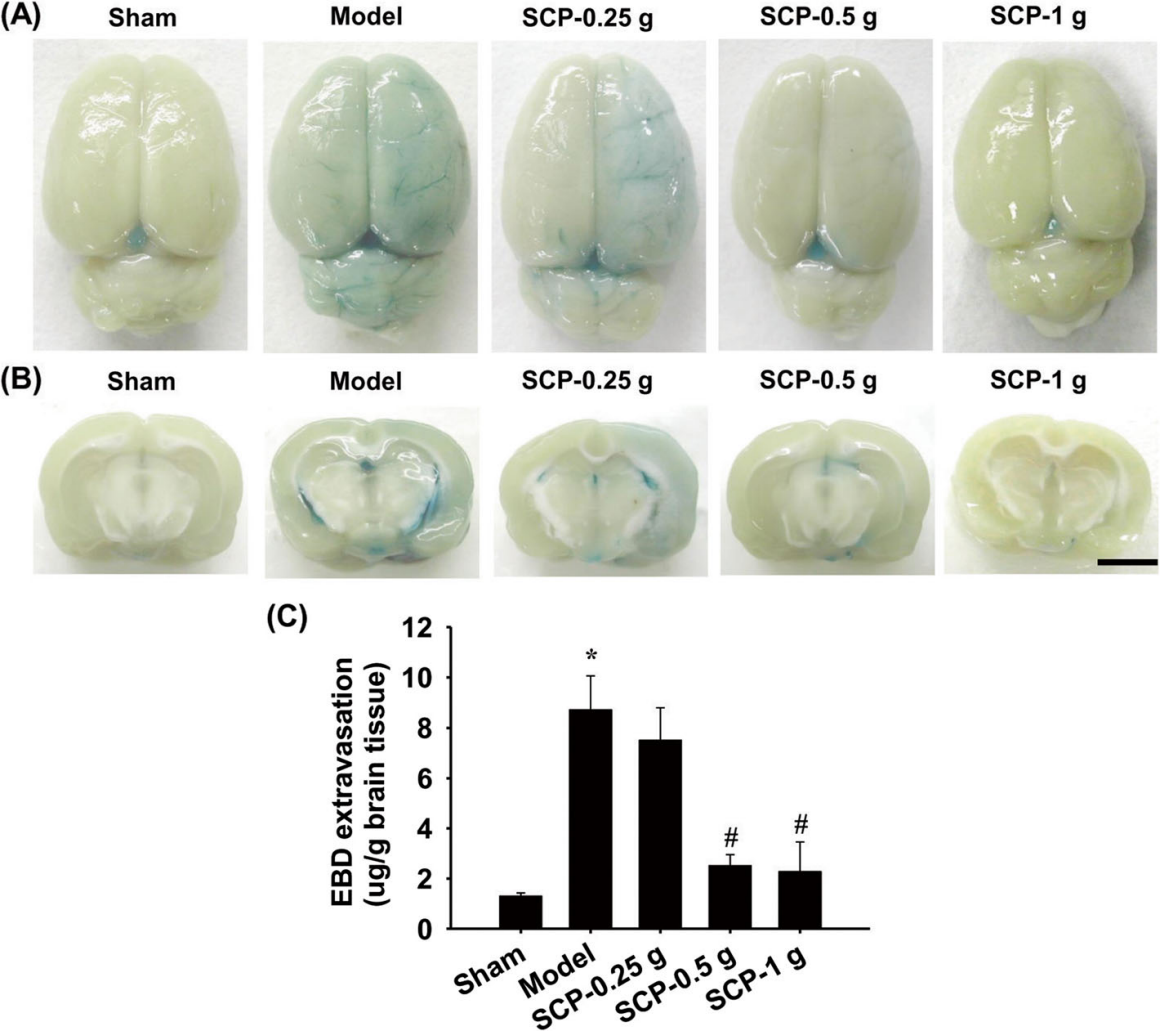
Effects of SCP-0.5 g and SCP-1 g treatments on BBB permeability in right cortical regions. Representative images (**a**) and (**b**) show EBD extravasation in right cortical regions in the Sham, Model, SCP-0.25 g, SCP-0.5 g, and SCP-1 g groups 3 d after reperfusion. **c** EBD extravasation was examined in the selected right cortical regions among the experimental groups. Scale bar = 5 mm. EBD, Evans blue dye. *P < 0.05 compared with the Sham group; #*P* < 0.05 compared with the Model group

BBB permeability in the selected right cortical regions markedly increased in the Model group (6.7-fold) compared with the Sham group (*P* < 0.05) and markedly decreased in the SCP-0.5 g (0.3-fold) and SCP-1 (0.3-fold) g groups compared with the Model group 3 d after reperfusion (both *P* < 0.05; Fig. 4a–c). However, no marked difference was found in BBB permeability in the selected right cortical regions between the Model and SCP-0.25 g groups (*P* > 0.05).

### Effects of SCP treatments on the cytosolic expression of GFAP, NKCC1, and p-JNK/JNK

**Fig. 5:**
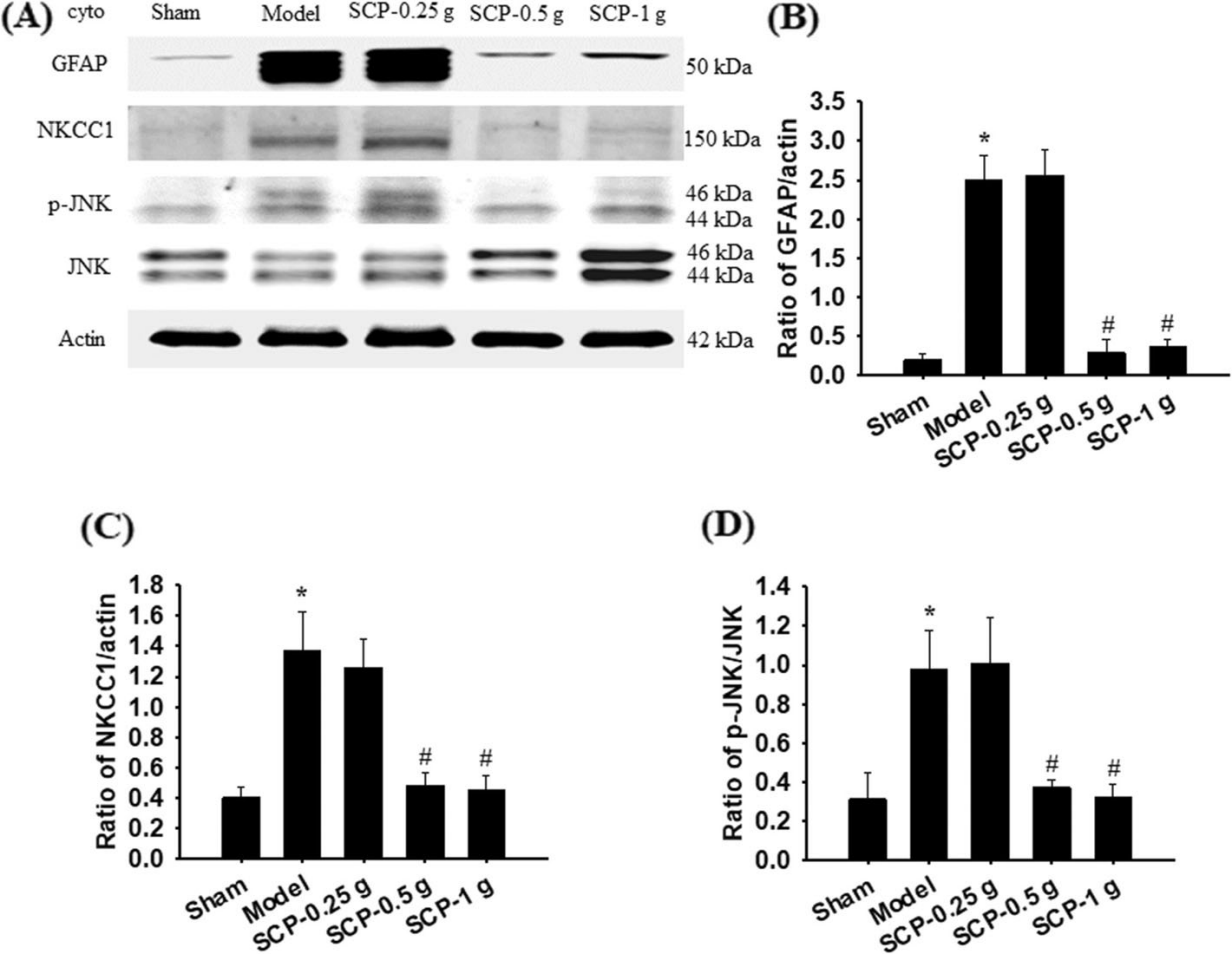
Effects of SCP-0.5 g and SCP-1 g treatments on cytosolic GFAP, NKCC1, p-JNK, and JNK expression in the penumbra of the cerebral cortex. **a** Representative images show cytosolic GFAP, NKCC1, p-JNK, and JNK expression in the penumbra of the cerebral cortex in the Sham, Model, SCP-0.25 g, SCP-0.5 g, and SCP-1 g groups 3 d after reperfusion. The ratios of **b** GFAP/actin, **c** NKCC1/actin, and **d** p-JNK/JNK expression were examined in the penumbra of the cerebral cortex among the experimental groups. Actin levels were used as internal controls. Cyto, cytosolic fraction. *P < 0.05 compared with the Sham group; #*P* < 0.05 compared with the Model group

The cytosolic expression of GFAP/actin, NKCC1/actin, and p-JNK/JNK in the penumbra of the cerebral cortex markedly increased in the Model group (13.2-, 3.4-, and 3.2-fold, respectively) compared with the Sham group (all *P* < 0.05) and markedly decreased in the SCP-0.5 g (0.1-, 0.4-, and 0.4-fold, respectively) and SCP-1 g (0.1-, 0.3-, and 0.3-fold) groups compared with the Model group 3 d after reperfusion (all *P* < 0.05; Fig. 5a–d). However, the aforementioned protein levels showed no marked difference between the Model and SCP-0.25 g groups (*P* > 0.05).

### Effects of SCP treatments on the cytosolic expression of iNOS, VEGF-A, ZO-1, ZO-2, and ZO-3

**Fig. 6:**
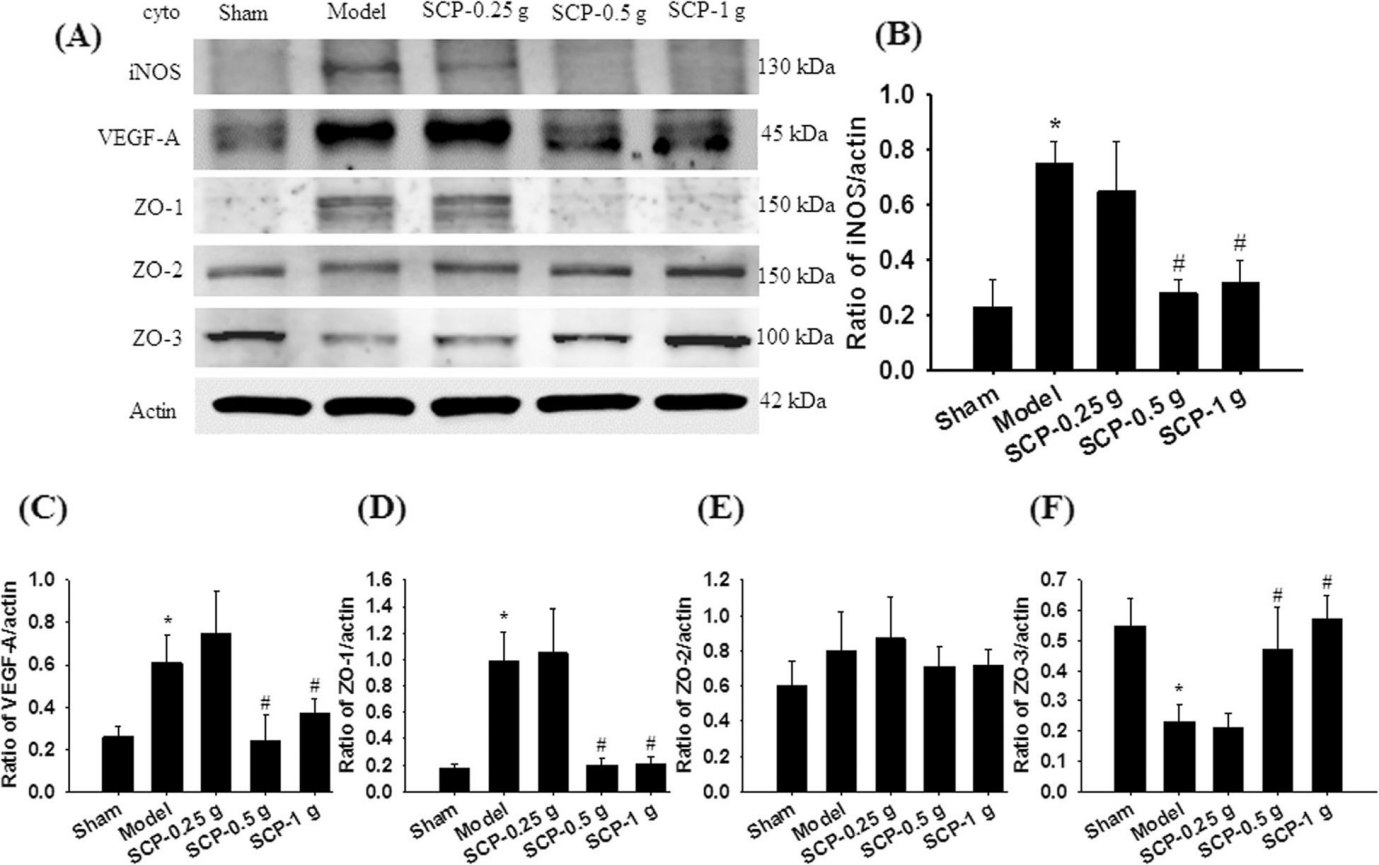
Effects of SCP-0.5 g and SCP-1 g treatments on cytosolic iNOS, VEGF-A, ZO-1, ZO-2, and ZO-3 expression in the penumbra of the cerebral cortex. **a** Representative images show cytosolic iNOS, VEGF-A, ZO-1, ZO-2, and ZO-3 expression in the penumbra of the cerebral cortex in the Sham, Model, SCP-0.25 g, SCP-0.5 g, and SCP-1 g groups 3 d after reperfusion. The ratios of **b** iNOS/actin, **c** VEGF-A/actin, **d** ZO-1/actin, **e** ZO-2/actin, and **f** ZO-3/actin expression were examined in the penumbra of the cerebral cortex among the experimental groups. *P < 0.05 compared with the Sham group; #*P* < 0.05 compared with the Model group

The cytosolic expression of iNOS/actin, VEGF-A/actin, and ZO-1/actin in the penumbra of the cerebral cortex markedly increased in the Model group (3.3-, 2.5-, and 5.5-fold, respectively) compared with the Sham group (all *P* < 0.05) and markedly decreased in the SCP-0.5 g (0.4-, 0.4-, and 0.2-fold, respectively) and SCP-1 g (0.4-, 0.6-, and 0.2-fold, respectively) groups compared with the Model group 3 d after reperfusion (all *P* < 0.05; Fig. 6a–d). By contrast, the cytosolic expression of ZO-3/actin in the penumbra of the cerebral cortex markedly decreased in the Model group (0.4-fold) compared with the Sham group (*P* < 0.05) and markedly increased in the SCP-0.5 g (2.0-fold) and SCP-1 g (2.5-fold) groups compared with the Model group (both *P* < 0.05; Fig. 6a and f). However, the aforementioned protein levels showed no marked difference between the Model and SCP-0.25 g groups (*P* > 0.05). The cytosolic expression of ZO-2/actin showed no marked difference among the experimental groups (*P* > 0.05; Fig. 6a and e).

### Effects of SCP treatments on AQP4, NKCC1, 3-NT, ICAM-1, ZO-1, and MMP-9 expression

**Fig. 7:**
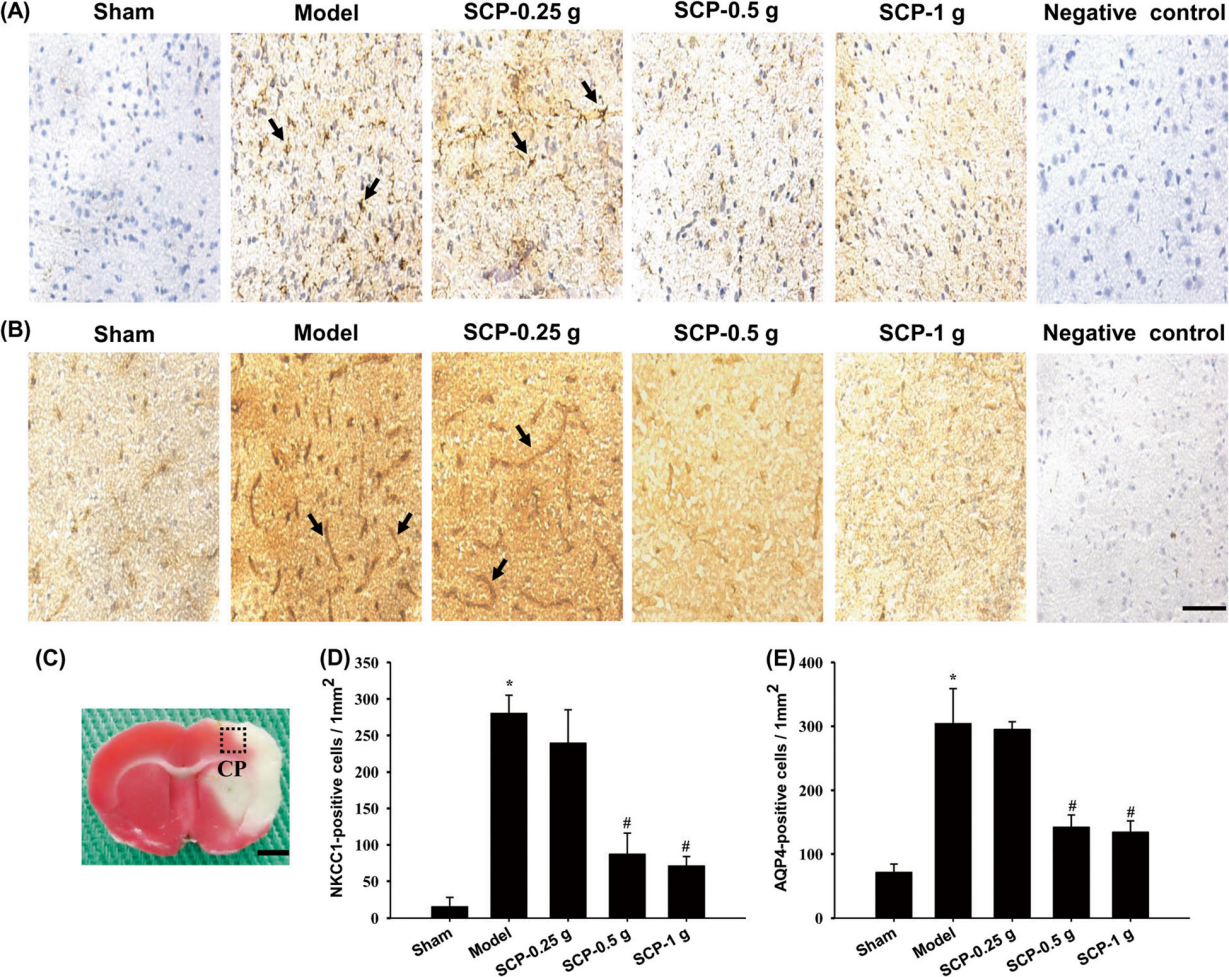
Effects of SCP-0.5 g and SCP-1 g treatments on NKCC1 and AQP4 expression in the penumbra of the cerebral cortex. Representative images show **a** NKCC1 and **b** AQP4 expression in the penumbra of the cerebral cortex in the Sham, Model, SCP-0.25 g, SCP-0.5 g, and SCP-1 g groups 3 d after reperfusion. **c** The dotted line square in a TTC-stained coronal brain section reveals the region in which the immunopositive cells are counted. CP, cortical penumbra. Dotted line square = 1 mm^2^. The bar graphs display the numbers of **d** NKCC1- and **e** AQP4-positive cells in the penumbra of the cerebral cortex among the experimental groups. *P < 0.05 compared with the Sham group; #*P* < 0.05 compared with the Model group. Arrows in (**a**) and (**b**) point to NKCC1- and AQP4-positive cells, respectively. Scale bars = 10 μm and 2 mm for (**b**) and (**c**), respectively

**Fig. 8:**
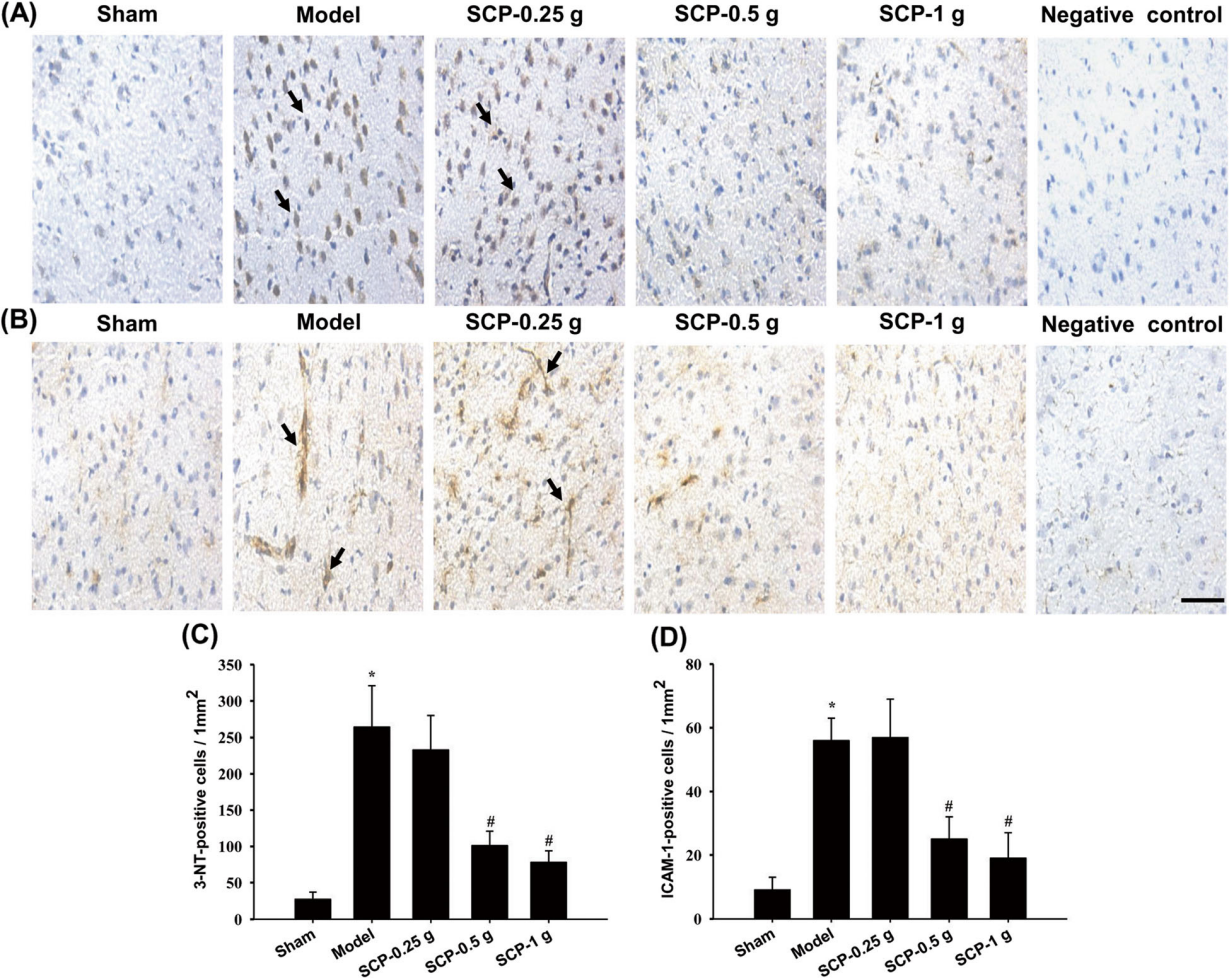
Effects of SCP-0.5 g and SCP-1 g treatments on 3-NT and ICAM-1 expression in the penumbra of the cerebral cortex. Representative images show **a** 3-NT and **b** ICAM-1 expression in the penumbra of the cerebral cortex in the Sham, Model, SCP-0.25 g, SCP-0.5 g, and SCP-1 g groups 3 d after reperfusion. The bar graphs display the numbers of **c** 3-NT- and **d** ICAM-1-positive cells in the penumbra of the cerebral cortex among the experimental groups. *P < 0.05 compared with the Sham group; #*P* < 0.05 compared with the Model group. Arrows in (**a**) and (**b**) point to 3-NT- and ICAM-1-positive cells, respectively. Scale bar = 10 μm

**Fig. 9:**
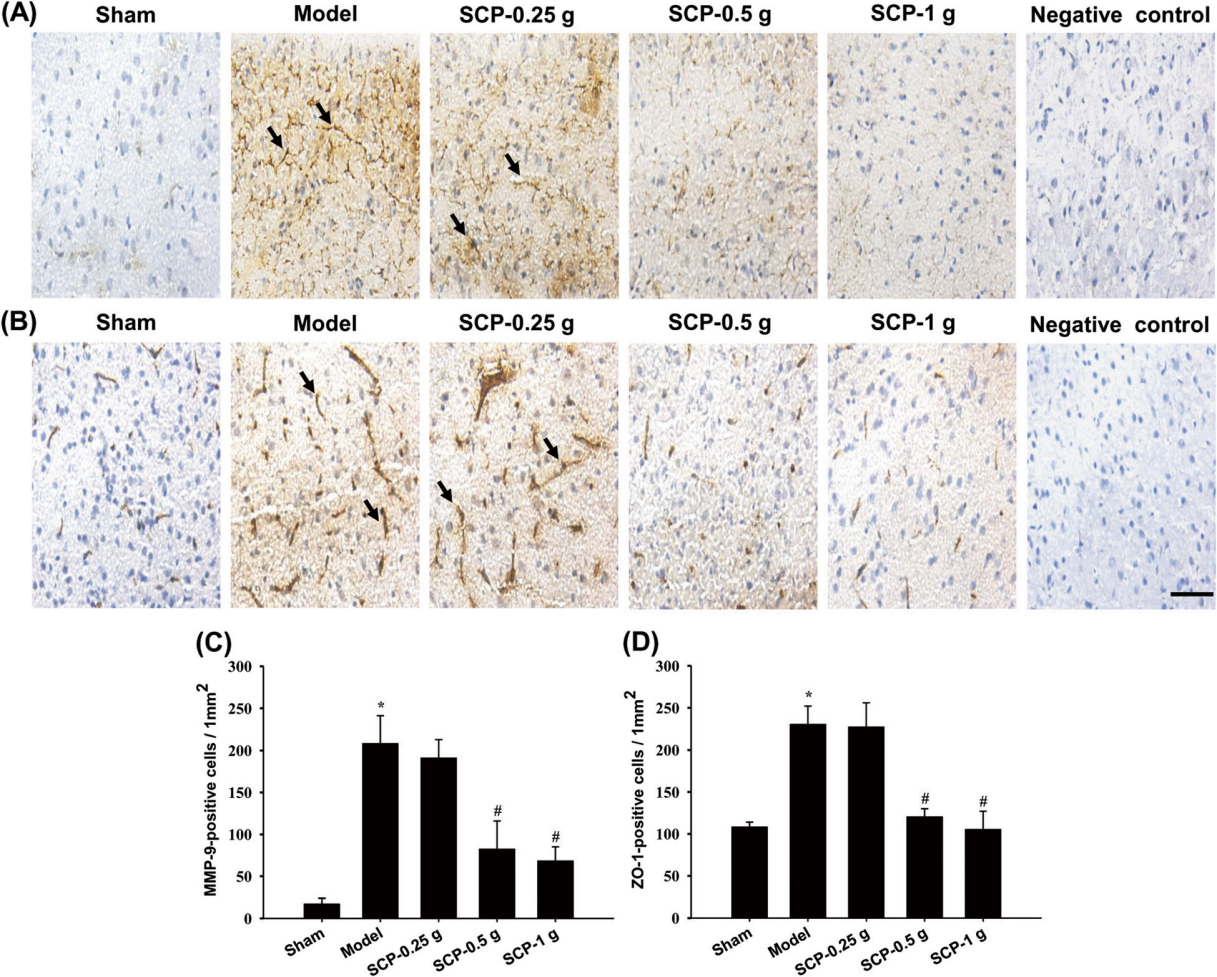
Effects of SCP-0.5 g and SCP-1 g treatments on MMP-9 and ZO-1 expression in the penumbra of the cerebral cortex. Representative images show **a** MMP-9 and **b** ZO-1 expression in the penumbra of the cerebral cortex in the Sham, Model, SCP-0.25 g, SCP-0.5 g, and SCP-1 g groups 3 d after reperfusion. The bar graphs display the numbers of **c** MMP-9- and **d** ZO-1-positive cells in the penumbra of the cerebral cortex among the experimental groups. *P < 0.05 compared with the Sham group; #*P* < 0.05 compared with the Model group. Arrows in (**a**) and (**b**) point to MMP-9- and ZO-1-positive cells, respectively. Scale bar = 10 μm

The numbers of AQP4-, NKCC1-, 3-NT-, ICAM-1-, ZO-1-, and MMP-9-positive cells were measured within the dotted line square in the penumbra of the cerebral cortex (counts/1 mm^2^; Fig. 7c) 3 d after reperfusion. The numbers of these immunopositive cells markedly increased in the Model groups compared with the Sham group (all *P* < 0.05) and markedly decreased in the SCP-0.5 g and SCP-1 g groups compared with the Model group (all *P* < 0.05; Figs. 7a, b, d, e, 8a–d, and 9a–d). However, the aforementioned immunopositive cells showed no marked difference between the Model and SCP-0.25 g groups (*P* > 0.05).

### Expression of GFAP- and NKCC1-positive cells and AQP4/NKCC1 and ICAM-1/ZO-1 double-labeled cells in the cortical penumbra

**Fig. 10:**
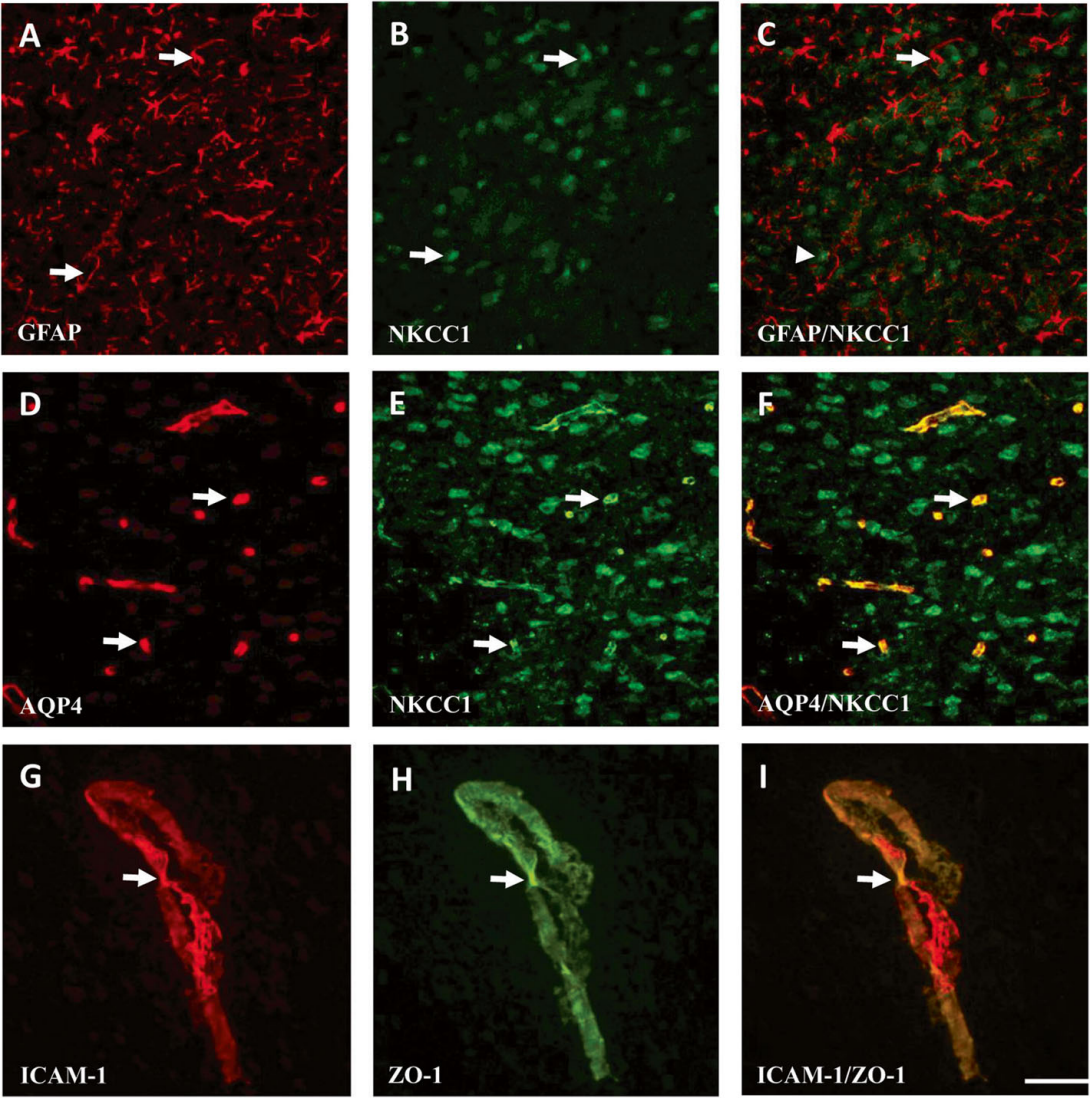
Expression of GFAP-, NKCC1-, AQP4-, ICAM-1-, and ZO-1-positive cells in the penumbra of the cerebral cortex. Arrows in (**a**), (**b**), (**d**), (**e**), (**g**), and (**h**) point to GFAP-, NKCC1-, AQP4-, NKCC1-, ICAM-1-, and ZO-1-positive cells, respectively, in the penumbra of the cerebral cortex. The arrow and arrowhead in (**c**) point to GFAP- and NKCC1-positive cells, respectively. Arrows in (**f**) and (**i**) point to AQP4/NKCC1 and ICAM-1/ZO-1 double-labeled cells, respectively. Scale bar = 50 μm

GFAP-, NKCC1-, and AQP4-immunopositive cells were predominantly observed in the penumbra of the cerebral cortex 3 d after reperfusion (Fig. 10a, b, d, and e). The expression levels of NKCC1 were positively correlated with those of GFAP (Fig. 10c). AQP4 was colocalized with NKCC1 in the cortical penumbra (Fig. 10d–f). In addition, ZO-1 was predominantly expressed in vascular endothelial cells and was colocalized with ICAM-1 (Fig. 10g–i).

## Discussion

Inflammatory responses and oxidative/nitrative stress are the main pathological characteristics of I/R-induced cerebral infarction, which is closely associated with BBB disruption and astrocyte activation; these are followed by cerebral edema in the early phase of ischemic stroke [1, 43, 44]. Previous studies have reported that cerebral edema initiated as early as 3–6 h and maintained at a high level until 72 h after reperfusion exacerbates cerebral infarction during the acute phase of focal cerebral ischemia [4, 45]. In this study, the TTC staining results revealed that prominent infarction occurred in the right cerebral hemisphere including the cortex and striatum 3 d after reperfusion. However, the SCP extract administered at doses of 0.5 g/kg (SCP-0.5 g) and 1 g/kg (SCP-1 g), but not 0.25 g/kg (SCP-0.25 g), markedly reduced cerebral areas and effectively restored neurological function. In addition, SCP-0.5 g and SCP-1 g treatments markedly reduced water content in the right cerebral hemisphere. During the early stage of cerebral I/R injury, the upregulated expression of reactive astrogliosis in the ischemic penumbra is closely associated with the extension of cerebral infarction and cerebral edema [13, 39, 42, 46]. Our Western blot results showed that the expression of GFAP, a marker of reactive astrocytes, was markedly elevated in the penumbra of the cerebral cortex 3 d after reperfusion, whereas SCP-0.5 g and SCP-1 g treatments effectively reduced the elevated expression of GFAP in the peri-infarct area. Based on these results, we suggest that SCP-0.5 g and SCP-1 g treatments effectively reduce cerebral infarction, improve neurological function, and ameliorate cerebral edema 3 d after reperfusion. Furthermore, the neuroprotective effects of SCP extract treatments against cerebral I/R injury are at least partially due to the inhibition of reactive astrocyte-mediated infarct expansion and cerebral edema in the early phase of transient MCAo.

Reactive astrocytes are important participants in cerebral edema due to overexpression of water transport proteins during cerebral I/R injury [13]. Under cerebral ischemic conditions, the deprivation of ATP and decreased activity of Na^+^/K^+^-ATPase results in the transport of Na^+^ ions into neurons and glial cells; subsequently, the entry of Na^+^ ions causes Cl^−^ ions influx through chloride channels, which leads to an increase in plasma osmolarity of cells [11, 47]. In addition, glutamate-induced elevated levels of extracellular K^+^ ions trigger NKCC1 activation, which moves one Na^+^, one K^+^, and two Cl^−^ ions into the astrocyte, enhancing the intracellular osmotic pressure and accompanying water influx [8]. AQP4, a water-specific transport protein, plays a crucial role in the movement of water into and out of the brain parenchyma. During MCAo, AQP4 is prominently expressed in the glial limitans and astrocyte endfeet covering the endothelial cells of the BBB, which subsequently facilities water passage into the astrocytes according to osmotic gradients, leading to astrocytic swelling (cytotoxic edema) within 24–72 h after the initiation of the cerebral ischemic insult [13, 48]. Pharmacological inhibition of NKCC1 or AQP4 expression in the ischemic area effectively reduced the infarct size and ameliorated cerebral edema in rat models of transient [49] and permanent [50, 51] MCAo. Our Western blot assay, immunohistochemical (IHC), and immunofluorescence (IF) double staining results revealed that AQP4 was colocalized with NKCC1, which was consistent with the elevation of GFAP expression in the cortical penumbra. Furthermore, the expression levels of NKCC1 and AQP4 were significantly increased in the penumbra of the cerebral cortex 3 d after reperfusion. However, SCP-0.5 g and SCP-1 g treatments effectively attenuated the expression of these proteins. The present findings indicate that the neuroprotective effects of SCP extract treatments on cerebral edema are partially due to the inhibition of astrocytic swelling in the cortical penumbra. Furthermore, the anti-cytotoxic edema effects of SCP extract treatments are partially attributed to the downregulation of astrocytic NKCC1/AQP4 signaling 3 d after reperfusion.

In cerebral I/R injury, NKCC1- and AQP4-mediated astrocytic swelling triggers JNK activation, which subsequently induces oxidative/nitrative stress and pro-inflammatory cytokine production, resulting in disruption of BBB integrity and exacerbation of cerebral edema [11, 12, 52]. JNK, one of the MAPK family members, participates in various cerebral I/R insults and plays a pivotal role in inflammation and apoptosis. Accumulated evidence indicates that the activation of JNK signaling triggers subsequent iNOS-induced NO overproduction during cerebral I/R injury [17, 53]. Large amounts of NO react rapidly with superoxide anions to produce high levels of peroxynitrite, which causes the nitration of tyrosine resides to form 3-NT (a footprint of peroxynitrite production) in the cell membrane and organelles [26]. The robust expression of iNOS and 3-NT is present in the ischemic area as early as 24 h and up to 96 h after cerebral reperfusion [16, 54]. Oxidative/nitrative stress causes the upregulation of adhesive molecules, such as ICAM-1, triggers leukocyte infiltration into the infarct area, and causes endothelial dysfunction, resulting in BBB disruption and initiating vasogenic edema formation in the early phase of ischemic stroke [15]. In addition, peroxynitrite-mediated oxidative/nitrative stress increases the expression of MMP-9 in endothelial cells, augmenting I/R-induced inflammatory responses and exacerbating BBB disruption [18]. Thus, a close relationship exists between oxidative/nitrative stress and MMP-9 in the pathology of BBB disruption. Furthermore, MMP-9 activation leads to degradation of TJs in endothelial cells and finally increases BBB permeability in the ischemic region [55]. VEGF-A, a VEGF family member, plays a key role in angiogenesis and vascular permeability. VEGF-A can regulate the expression of components of TJ proteins in endothelial cells through the activation of MMPs, and it is positively associated with BBB disruption and cerebral edema in the acute phase of cerebral ischemia [24, 39]. Previous studies have reported that vasogenic edema occurs as early as 4 h and reaches a peak at 24–72 h after the onset of ischemia [18, 55]. Therapeutic agents that inhibit JNK activation and reduce iNOS, 3-NT, ICAM-1, MMP-9, and VEGF expression in the ischemic region effectively restore BBB integrity, reduce the cerebral infarct area, and ameliorate vasogenic edema in rat models of cerebral I/R injury [15, 16, 18, 24, 55]. Brain water content and BBB permeability can be assessed as the indicators of vasogenic edema induced by I/R injury [56]. In the present study, water content and BBB permeability in the selected ischemic regions were markedly increased 3 d after reperfusion, a finding consistent with those of the aforementioned studies [18, 55]. However, SCP-0.5 g and SCP-1 g treatments effectively restored water content and BBB permeability in the selected ischemic regions. Moreover, our Western blot and IHC findings showed that the expression levels of p-JNK/JNK ratio, iNOS, 3-NT, ICAM-1, MMP-9, and VEGF-A were markedly upregulated in the penumbra of the cerebral cortex, whereas SCP extract treatments effectively downregulated the expression of the aforementioned proteins 3 d after reperfusion. On the basis of these results, we suggest that SCP extract treatments protect against cerebral I/R-induced BBB damage partially by downregulating the astrocytic swelling-mediated JNK/iNOS signaling pathway in the cortical penumbra. Furthermore, the anti-vasogenic edema effects of SCP extract treatments are partially due to the downregulation of JNK/iNOS-mediated ICAM-1/MMP-9/VEGF-A signaling 3 d after reperfusion.

Previous studies have demonstrated that the increase in BBB permeability and the disruption of BBB integrity are positively related to cerebral edema [3, 24]. The BBB is composed of endothelial cells that represent capillaries in the brain parenchyma, and TJ offers an effective barrier between the endothelial cells [57]. The main proteins embedded in the membrane of the endothelial TJ are claudins, occludin, and ZO [58]. Cytoplasmic TJ accessory proteins including ZO-1, − 2, and − 3 form and stabilize the TJ barrier by connecting occludin to the actin cytoskeleton [59]. Furthermore, in the molecular structure of TJ proteins, ZO-1/ZO-2 and ZO-1/ZO-3 heterodimers are directly involved in the regulation of the barrier function of TJs [60]. A previous study reported that ZO-1 plays dual roles in the promotion of TJ integrity and endothelial chemotaxis in an in vitro model of sphingosine-1-phosphate–treated vascular endothelial cells [61]. Zan et al. (2014) conduced the time-course expression analysis of the TJ protein and revealed that the expression of ZO-1 mRNA and protein was significantly decreased in the ischemic area 1 d but gradually increased 3–7 d after cerebral I/R [62]. In addition, previous results revealed that pharmacological upregulation of ZO-1 expression exerts neuroprotective effects against cerebral infarction and cerebral edema by preserving endothelial TJ integrity in the ischemic area 1 d after reperfusion [3, 57, 62]. The results of this study showed that the expression of ZO-1 markedly increased in the cortical penumbra, whereas the expression of ZO-3 significantly decreased 3 d after reperfusion. However, SCP-0.5 g and SCP-1 g treatments effectively reversed ZO-1 and ZO-3 but did not affect ZO-2 expression in the cortical penumbra 3 d after reperfusion. Moreover, our Western blot, IHC, and IF double staining data revealed that ZO-1 was colocalized with ICAM-1, and the expression patterns of ZO-1 in the experimental groups were consistent with those of ICAM-1/MMP-9/VEGF-A signaling. The aforementioned results suggest that ZO-1 and ZO-3 play roles in the enhancement of endothelial chemotaxis and maintenance of TJ integrity, respectively in the cortical penumbra 3 d after reperfusion. In the initial stage of cerebral I/R injury, endothelial TJ disruption is partially induced by activating ICAM-1/MMP-9/VEGF-A signaling in the ischemic area. Later, this cascade triggers ZO-1 overexpression, which further elicits the chemotactic activity of leukocyte infiltration into endothelial cells, exacerbating cerebral edema. Further analysis indicates that the effects of SCP extract treatments on vasogenic edema are partially mediated by the downregulation of JNK/iNOS-mediated ICAM-1/MMP-9/VEGF-A/ZO-1 and the upregulation of ZO-3 signaling in the penumbra of cerebral cortex 3 d after reperfusion. To our knowledge, this is the first study to report the positive relationship between ZO-1 and ICAM-1 expression in vascular endothelial cells in the ischemic area following cerebral I/R injury. However, the precise mechanisms underlying TJ integrity preservation by ZO-3 still need to be elucidated.

## Conclusions

Taken together, the study results revealed that the SCP extract administered at doses of 0.5 g/kg and 1 g/kg markedly reduced cerebral infarction, alleviated cerebral edema, and restored neurological function in the early phase of transient MCAo. The effects of SCP extract treatments on cerebral I/R injury-induced cerebral edema are partially due to the inhibition of astrocytic swelling and BBB disruption in the cortical penumbra. Furthermore, the anti-edema effects of SCP extract treatments are possibly associated with the downregulation of astrocytic NKCC1/AQP4 and JNK/iNOS-mediated ICAM-1/MMP-9 signaling in the penumbra of the cerebral cortex 3 d after reperfusion. Thus, the results of this study indicate that the *A. tatarinowii* Schott extract can be a therapeutic agent for reducing cerebral edema after cerebral I/R injury. However, further research is required to elucidate precise mechanisms underlying the anti-cerebral edema effects of the *A. tatarinowii* Schott extract for future clinical applications.

## Data Availability

The datasets used and/or analysed during the current study are available from the corresponding author on reasonable request.

## Abbreviations

SCP: *Acorus tatarinowii* Schott (Shi Chang Pu)
MCAo: Middle cerebral artery occlusion
KCC1: Na^+^-K^+^-2Cl^−^ cotransporter type 1
AQP4: Aquaporin 4
JNK: c-Jun N-terminal kinase
iNOS: Inducible nitric oxide synthase
ICAM-1: Intercellular adhesion molecule-1
MMP-9: Matrix metalloproteinase-9
VEGF-A: Vascular endothelial growth factor-A
ZO-1: Zonula occluden-1
BBB: Blood–brain barrier
I/R: Ischemia–reperfusion
NO: Nitric oxide
GFAP: Glial fibrillary acidic protein
MAPK: Mitogen-activated protein kinase
iNOS: Inducible nitric oxide synthase
3-NT: 3-nitrotyrosine
TJ: Tight junction
HPLC: High performance liquid chromatography
MCA: Middle cerebral artery
ECA: External carotid artery
ICA: Internal carotid artery
mNSS: Modified neurological severity score
TTC: 2,3,5-triphenyltetrazolium chloride
EBD: Evans blue dye
RT: Room temperature
NC: Nitrocellulose
IHC: Immunohistochemical
IF: Immunofluorescence
